# Hæmorrhage from the Gums

**Published:** 1864-03

**Authors:** M. Limousin


Hæmorrhage from the Gums.—It is often extremely difficult to restrain the hæmorrhage consequent on the extraction of a tooth. I was recently consulted by a young woman suffering from an accident of this description: she was deadly pale, faint, and threw up, without cessation, mouthfuls of blood, which issued from the alveolar of the second large molar, which had been extracted without difficulty. Sesquichloride of iron and sulphuric acid had in vain been resorted to. I applied over the alveolar a small pyramid of discs of agaric, piled up sufficiently high to rise above the level of the adjacent teeth, so that the mere act of closing the jaws caused powerful pressure on the seat of the hæmorrhage. The escape of blood was immediately checked, and did not return in the morning when the dressing was removed. I may also be permitted to allude to an equally simple and efficacious method of arresting the flow of blood from leech-bites. This can always be effected by slight pressure with the tip of the finger. This procedure is invariably successful, and may be safely entrusted to the watchful care of a mother. M. Limousin, M.D. —*Journal of Practical Medicine and Surgery.*

